# Clinical Treatise on the Pathology and Therapeuty of Disorders of Metabolism and Nutrition

**Published:** 1904-03

**Authors:** 


Clinical Treatise on the Pathology and Therapeuty of Disorders of Metabolism and Nutrition. By Prof. Dr. Carl von Noorden, Physician-in-Chief to the City Hospital, Frankfort a. M.

The volume under consideration is part II, devoted to *nephritis*. It is the authorized American edition translated under the direction of Dr. Boardman Reed, of Philadelphia. E. B. Treat & Co., of New York, is the publisher. Price, $1.00. Part 1 of the series is on *obesity, the indications for reduction cures.* Price, 50 cents. These are along basic lines, and in many respects they are iconoclastic.

